# *N*-(2,6-Dimethyl­phen­yl)-4-methyl­benzene­sulfonamide

**DOI:** 10.1107/S1600536810014571

**Published:** 2010-04-24

**Authors:** P. G. Nirmala, B. Thimme Gowda, Sabine Foro, Hartmut Fuess

**Affiliations:** a Department of Chemistry, Mangalore University, Mangalagangotri 574 199, Mangalore, India; b Institute of Materials Science, Darmstadt University of Technology, Petersenstrasse 23, D-64287 Darmstadt, Germany

## Abstract

In the title compound, C_15_H_17_NO_2_S, the mol­ecule is bent at the S atom, the C—SO_2_—NH—C torsion angle being 88.0 (2)°. The dihedral angle between the two aromatic rings is 49.8 (1)°. In the crystal, mol­ecules are linked into zigzag chains parallel to the *a* axis *via* N—H⋯O hydrogen bonds.

## Related literature

For the preparation of the title compound, see: Shetty & Gowda (2005 ▶). For our study of the effect of substituents on the structures of *N*-(ar­yl)-aryl­sulfonamides, see: Gowda *et al.* (2008 ▶, 2009 ▶, 2010 ▶). For related structures, see: Gelbrich *et al.* (2007 ▶); Perlovich *et al.* (2006 ▶).
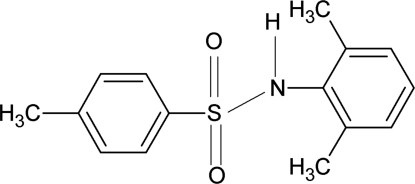


## Experimental

### 

#### Crystal data

C_15_H_17_NO_2_S
*M* *_r_* = 275.36
Monoclinic, 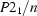

*a* = 5.1412 (5) Å
*b* = 17.310 (2) Å
*c* = 16.429 (2) Å
β = 96.65 (1)°
*V* = 1452.2 (3) Å^3^
*Z* = 4
Mo *K*α radiation
μ = 0.22 mm^−1^
*T* = 299 K
0.46 × 0.32 × 0.14 mm

#### Data collection

Oxford Diffraction Xcalibur diffractometer with a Sapphire CCD Detector.
Absorption correction: multi-scan (*CrysAlis RED*; Oxford Diffraction, 2009 ▶) *T*_min_ = 0.906, *T*_max_ = 0.970
7741 measured reflections
2580 independent reflections
2090 reflections with *I* > 2σ(*I*)
*R*_int_ = 0.052

#### Refinement

*R*[*F* ^2^ > 2σ(*F* ^2^)] = 0.042
*wR*(*F* ^2^) = 0.116
*S* = 1.04
2580 reflections
178 parameters
1 restraint
H atoms treated by a mixture of independent and constrained refinement
Δρ_max_ = 0.25 e Å^−3^
Δρ_min_ = −0.36 e Å^−3^

### 

Data collection: *CrysAlis CCD* (Oxford Diffraction, 2009 ▶); cell refinement: *CrysAlis RED* (Oxford Diffraction, 2009 ▶); data reduction: *CrysAlis RED*; program(s) used to solve structure: *SHELXS97* (Sheldrick, 2008 ▶); program(s) used to refine structure: *SHELXL97* (Sheldrick, 2008 ▶); molecular graphics: *PLATON* (Spek, 2009 ▶); software used to prepare material for publication: *SHELXL97*.

## Supplementary Material

Crystal structure: contains datablocks I, global. DOI: 10.1107/S1600536810014571/vm2025sup1.cif

Structure factors: contains datablocks I. DOI: 10.1107/S1600536810014571/vm2025Isup2.hkl

Additional supplementary materials:  crystallographic information; 3D view; checkCIF report

## Figures and Tables

**Table 1:** Hydrogen-bond geometry (Å, °)

*D*—H⋯*A*	*D*—H	H⋯*A*	*D*⋯*A*	*D*—H⋯*A*
N1—H1*N*⋯O1^i^	0.85 (1)	2.20 (1)	3.040 (2)	169 (2)

Symmetry code: (i) 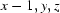.

**Fig. 1.**
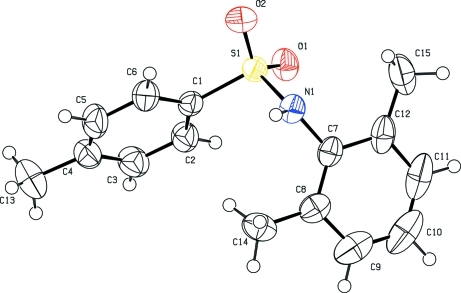
Molecular structure of (I), showing the atom labelling scheme and displacement ellipsoids are drawn at the 50% probability level.

**Fig. 2.**
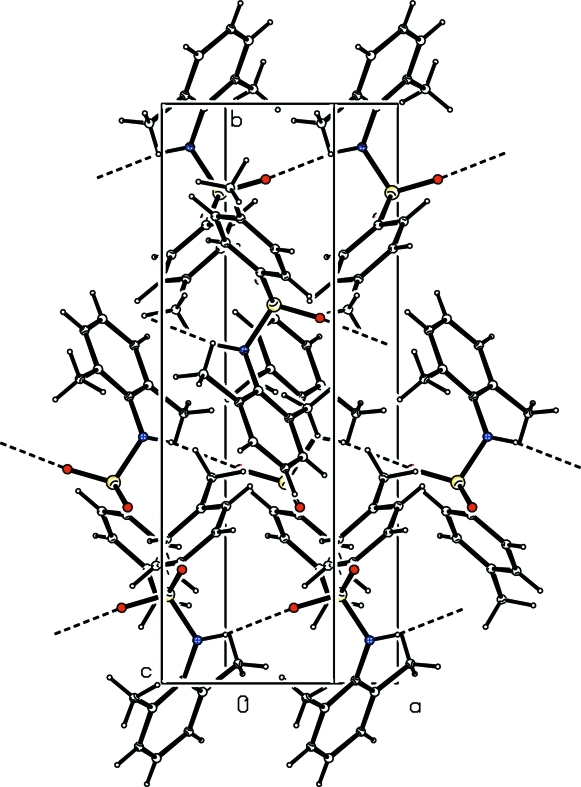
Molecular packing of (I) with hydrogen bonding shown as dashed lines.

## supplementary crystallographic information

### Comment

In the present work, as part of a study of the effect of substituents on the crystal structures of *N*-(aryl)-arylsulfonamides (Gowda *et al.*, 2008, 2009, 2010), the structure of *N*-(2,6-dimethylphenyl)-4-methylbenzenesulfonamide (I) has been determined. The molecule is bent at the *S* atom (Fig. 1) with the C1—SO_2_—NH—C7 torsion angle of 88.0 (2)°, compared to the values of -51.6 (3)° in *N*-(phenyl)4-methylbenzenesulfonamide (II) (Gowda *et al.*, 2009), -78.7 (2)° in *N*-(2,6-dimethylphenyl)- benzenesulfonamide (III) (Gowda *et al.*, 2008) and -61.0 (2)° in *N*-(2,5-dimethylphenyl)-4-methylbenzenesulfonamide (IV), -61.8 (2)° in *N*-(3,4-dimethylphenyl)-4-methylbenzenesulfonamide (V) and 56.8 (2)° in *N*-(3,5-dimethylphenyl)-4-methylbenzenesulfonamide (VI)(Gowda *et al.*, 2010).

The two benzene rings in (I) are tilted relative to each other by 49.8 (1)°, compared to the values of 68.4 (1)° in (II), 44.9 (1)° in (III), 49.4 (1)° in (IV), 47.8 (1)° in (V) and 53.9 (1)° in (VI). The other bond parameters are similar to those observed in (II), (III), (IV), (V), (VI) and other aryl sulfonamides (Perlovich *et al.*, 2006; Gelbrich *et al.*, 2007).

In the crystal structure, the intermolecular N–H···O hydrogen bonds (Table 1) link the molecules into infinite zig-zag chains running parallel to the *a*-axis. Part of the crystal structure is shown in Fig. 2.

### Experimental

4-Methylbenzenesulfonylchloride was obtained by treating the solution of toluene (10 ml) in chloroform (40 ml) with chlorosulfonic acid (25 ml) by the procedure reported earlier (Gowda *et al.*, 2010). 4-Methylbenzenesulfonylchloride was then treated with 2,6-dimethylaniline in the stoichiometric ratio to obtain *N*-(2,6-dimethylphenyl)- 4-methylbenzenesulfonamide. The latter was recrystallized to constant melting point (110 °C) from dilute ethanol. The purity of the compound was checked and characterized by recording its infrared and NMR spectra (Shetty & Gowda, 2005).

The prism like single crystals used in X-ray diffraction studies were grown in ethanolic solution by a slow evaporation at room temperature.

### Refinement

The H atom of the NH group was located in a difference map and later restrained to N—H = 0.86 (1) Å. The other H atoms were positioned with idealized geometry using a riding model with C—H = 0.93–0.96 Å. All H atoms were refined with isotropic displacement parameters (set to 1.2 times of the *U*_eq_ of the parent atom).

### Crystal data

**Table:**

C_15_H_17_NO_2_S	*F*(000) = 584
*M**_r_* = 275.36	*D*_x_ = 1.259 Mg m^−^^3^
Monoclinic, *P*2_1_/*n*	Mo *K*α radiation, λ = 0.71073 Å
Hall symbol: -P 2yn	Cell parameters from 2102 reflections
*a* = 5.1412 (5) Å	θ = 2.5–27.7°
*b* = 17.310 (2) Å	µ = 0.22 mm^−^^1^
*c* = 16.429 (2) Å	*T* = 299 K
β = 96.65 (1)°	Prism, colourless
*V* = 1452.2 (3) Å^3^	0.46 × 0.32 × 0.14 mm
*Z* = 4	

### Data collection

**Table:**

Oxford Diffraction Xcalibur diffractometer with a Sapphire CCD Detector.	2580 independent reflections
Radiation source: fine-focus sealed tube	2090 reflections with *I* > 2σ(*I*)
graphite	*R*_int_ = 0.052
Rotation method data acquisition using ω and phi scans.	θ_max_ = 25.4°, θ_min_ = 2.5°
Absorption correction: multi-scan (*CrysAlis RED*; Oxford Diffraction, 2009)	*h* = −6→6
*T*_min_ = 0.906, *T*_max_ = 0.970	*k* = −20→20
7741 measured reflections	*l* = −18→19

### Refinement

**Table:**

Refinement on *F*^2^	Primary atom site location: structure-invariant direct methods
Least-squares matrix: full	Secondary atom site location: difference Fourier map
*R*[*F*^2^ > 2σ(*F*^2^)] = 0.042	Hydrogen site location: inferred from neighbouring sites
*wR*(*F*^2^) = 0.116	H atoms treated by a mixture of independent and constrained refinement
*S* = 1.04	*w* = 1/[σ^2^(*F*_o_^2^) + (0.0536*P*)^2^ + 0.6128*P*] where *P* = (*F*_o_^2^ + 2*F*_c_^2^)/3
2580 reflections	(Δ/σ)_max_ = 0.011
178 parameters	Δρ_max_ = 0.25 e Å^−^^3^
1 restraint	Δρ_min_ = −0.35 e Å^−^^3^

### Special details

**Table:**

Experimental. CrysAlis RED (Oxford Diffraction, 2009) Empirical absorption correction using spherical harmonics, implemented in SCALE3 ABSPACK scaling algorithm.
Geometry. All esds (except the esd in the dihedral angle between two l.s. planes) are estimated using the full covariance matrix. The cell esds are taken into account individually in the estimation of esds in distances, angles and torsion angles; correlations between esds in cell parameters are only used when they are defined by crystal symmetry. An approximate (isotropic) treatment of cell esds is used for estimating esds involving l.s. planes.
Refinement. Refinement of *F*^2^ against ALL reflections. The weighted *R*-factor wR and goodness of fit *S* are based on *F*^2^, conventional *R*-factors *R* are based on *F*, with *F* set to zero for negative *F*^2^. The threshold expression of *F*^2^ > σ(*F*^2^) is used only for calculating *R*-factors(gt) etc. and is not relevant to the choice of reflections for refinement. *R*-factors based on *F*^2^ are statistically about twice as large as those based on *F*, and *R*- factors based on ALL data will be even larger.

### Fractional atomic coordinates and isotropic or equivalent isotropic displacement parameters (Å^2^)

**Table:**

	*x*	*y*	*z*	*U*_iso_*/*U*_eq_	
C1	0.0076 (4)	0.79081 (11)	0.32294 (13)	0.0373 (5)	
C2	0.1791 (5)	0.79695 (14)	0.39383 (14)	0.0499 (6)	
H2	0.3173	0.8319	0.3971	0.060*	
C3	0.1426 (5)	0.75041 (15)	0.45988 (15)	0.0571 (6)	
H3	0.2573	0.7546	0.5078	0.069*	
C4	−0.0596 (5)	0.69793 (14)	0.45655 (15)	0.0537 (6)	
C5	−0.2303 (5)	0.69408 (15)	0.38508 (17)	0.0580 (7)	
H5	−0.3706	0.6599	0.3821	0.070*	
C6	−0.1976 (4)	0.73955 (13)	0.31845 (15)	0.0487 (5)	
H6	−0.3133	0.7357	0.2707	0.058*	
C7	−0.0218 (4)	0.99486 (12)	0.27900 (14)	0.0416 (5)	
C8	−0.1053 (4)	1.01478 (14)	0.35381 (15)	0.0499 (6)	
C9	−0.0115 (6)	1.08314 (17)	0.39028 (19)	0.0747 (8)	
H9	−0.0666	1.0982	0.4398	0.090*	
C10	0.1617 (7)	1.12894 (18)	0.3544 (3)	0.0913 (11)	
H10	0.2274	1.1736	0.3807	0.110*	
C11	0.2372 (6)	1.10901 (16)	0.2804 (3)	0.0823 (10)	
H11	0.3537	1.1407	0.2567	0.099*	
C12	0.1440 (5)	1.04222 (13)	0.23938 (18)	0.0575 (7)	
C13	−0.0937 (7)	0.6450 (2)	0.5270 (2)	0.0861 (10)	
H13A	−0.0207	0.6687	0.5773	0.103*	
H13B	−0.0054	0.5970	0.5198	0.103*	
H13C	−0.2768	0.6353	0.5289	0.103*	
C14	−0.2972 (5)	0.96625 (16)	0.39423 (16)	0.0607 (7)	
H14A	−0.2435	0.9131	0.3945	0.073*	
H14B	−0.4683	0.9712	0.3644	0.073*	
H14C	−0.3021	0.9836	0.4495	0.073*	
C15	0.2180 (6)	1.02526 (17)	0.1559 (2)	0.0785 (9)	
H15A	0.0714	1.0027	0.1228	0.094*	
H15B	0.3626	0.9898	0.1603	0.094*	
H15C	0.2675	1.0724	0.1309	0.094*	
N1	−0.1189 (3)	0.92553 (10)	0.23762 (11)	0.0406 (4)	
H1N	−0.281 (2)	0.9160 (14)	0.2363 (14)	0.049*	
O1	0.3230 (3)	0.86960 (9)	0.24338 (10)	0.0492 (4)	
O2	−0.0551 (3)	0.80389 (9)	0.16625 (10)	0.0554 (4)	
S1	0.05387 (10)	0.84674 (3)	0.23607 (3)	0.03851 (18)	

### Atomic displacement parameters (Å^2^)

**Table:**

	*U*^11^	*U*^22^	*U*^33^	*U*^12^	*U*^13^	*U*^23^
C1	0.0356 (11)	0.0335 (10)	0.0442 (11)	0.0020 (9)	0.0100 (8)	−0.0017 (9)
C2	0.0488 (13)	0.0495 (13)	0.0513 (13)	−0.0121 (11)	0.0052 (10)	0.0000 (11)
C3	0.0612 (15)	0.0641 (15)	0.0454 (13)	−0.0033 (13)	0.0033 (11)	0.0023 (12)
C4	0.0602 (15)	0.0513 (14)	0.0529 (14)	0.0018 (12)	0.0212 (11)	0.0051 (11)
C5	0.0527 (14)	0.0510 (14)	0.0727 (17)	−0.0149 (12)	0.0175 (12)	0.0064 (13)
C6	0.0434 (12)	0.0475 (12)	0.0550 (14)	−0.0075 (11)	0.0049 (10)	0.0010 (11)
C7	0.0352 (11)	0.0332 (10)	0.0554 (13)	0.0062 (9)	0.0011 (9)	0.0055 (9)
C8	0.0462 (13)	0.0463 (13)	0.0551 (14)	0.0088 (11)	−0.0037 (10)	−0.0043 (11)
C9	0.081 (2)	0.0580 (16)	0.080 (2)	0.0120 (16)	−0.0092 (16)	−0.0198 (15)
C10	0.090 (2)	0.0443 (16)	0.132 (3)	−0.0081 (16)	−0.018 (2)	−0.0160 (19)
C11	0.0689 (19)	0.0416 (15)	0.136 (3)	−0.0097 (14)	0.0094 (19)	0.0171 (18)
C12	0.0478 (14)	0.0377 (12)	0.0872 (19)	0.0043 (11)	0.0088 (12)	0.0190 (12)
C13	0.103 (2)	0.090 (2)	0.0696 (19)	−0.0100 (19)	0.0280 (17)	0.0248 (17)
C14	0.0623 (16)	0.0715 (17)	0.0504 (14)	0.0100 (13)	0.0157 (11)	−0.0022 (13)
C15	0.082 (2)	0.0637 (17)	0.098 (2)	0.0144 (15)	0.0422 (17)	0.0360 (17)
N1	0.0308 (8)	0.0399 (9)	0.0509 (10)	−0.0004 (8)	0.0039 (8)	0.0021 (8)
O1	0.0343 (8)	0.0494 (9)	0.0662 (10)	0.0025 (7)	0.0162 (7)	0.0091 (8)
O2	0.0681 (11)	0.0544 (10)	0.0442 (9)	−0.0033 (8)	0.0087 (7)	−0.0100 (8)
S1	0.0364 (3)	0.0375 (3)	0.0431 (3)	0.0002 (2)	0.0108 (2)	−0.0002 (2)

### Geometric parameters (Å, °)

**Table:**

C1—C6	1.374 (3)	C10—C11	1.362 (5)
C1—C2	1.381 (3)	C10—H10	0.9300
C1—S1	1.763 (2)	C11—C12	1.395 (4)
C2—C3	1.382 (3)	C11—H11	0.9300
C2—H2	0.9300	C12—C15	1.495 (4)
C3—C4	1.377 (3)	C13—H13A	0.9600
C3—H3	0.9300	C13—H13B	0.9600
C4—C5	1.384 (4)	C13—H13C	0.9600
C4—C13	1.503 (4)	C14—H14A	0.9600
C5—C6	1.374 (3)	C14—H14B	0.9600
C5—H5	0.9300	C14—H14C	0.9600
C6—H6	0.9300	C15—H15A	0.9600
C7—C8	1.391 (3)	C15—H15B	0.9600
C7—C12	1.397 (3)	C15—H15C	0.9600
C7—N1	1.440 (3)	N1—S1	1.6294 (18)
C8—C9	1.387 (4)	N1—H1N	0.848 (10)
C8—C14	1.507 (3)	O1—S1	1.4308 (15)
C9—C10	1.375 (5)	O2—S1	1.4251 (16)
C9—H9	0.9300		
			
C6—C1—C2	120.4 (2)	C12—C11—H11	119.2
C6—C1—S1	119.08 (17)	C11—C12—C7	117.1 (3)
C2—C1—S1	120.50 (16)	C11—C12—C15	119.8 (3)
C1—C2—C3	119.1 (2)	C7—C12—C15	123.1 (2)
C1—C2—H2	120.5	C4—C13—H13A	109.5
C3—C2—H2	120.5	C4—C13—H13B	109.5
C4—C3—C2	121.6 (2)	H13A—C13—H13B	109.5
C4—C3—H3	119.2	C4—C13—H13C	109.5
C2—C3—H3	119.2	H13A—C13—H13C	109.5
C3—C4—C5	117.9 (2)	H13B—C13—H13C	109.5
C3—C4—C13	121.8 (3)	C8—C14—H14A	109.5
C5—C4—C13	120.3 (2)	C8—C14—H14B	109.5
C6—C5—C4	121.6 (2)	H14A—C14—H14B	109.5
C6—C5—H5	119.2	C8—C14—H14C	109.5
C4—C5—H5	119.2	H14A—C14—H14C	109.5
C1—C6—C5	119.4 (2)	H14B—C14—H14C	109.5
C1—C6—H6	120.3	C12—C15—H15A	109.5
C5—C6—H6	120.3	C12—C15—H15B	109.5
C8—C7—C12	122.2 (2)	H15A—C15—H15B	109.5
C8—C7—N1	119.88 (19)	C12—C15—H15C	109.5
C12—C7—N1	117.8 (2)	H15A—C15—H15C	109.5
C9—C8—C7	117.8 (3)	H15B—C15—H15C	109.5
C9—C8—C14	119.8 (2)	C7—N1—S1	123.04 (13)
C7—C8—C14	122.4 (2)	C7—N1—H1N	117.4 (16)
C10—C9—C8	121.1 (3)	S1—N1—H1N	111.9 (17)
C10—C9—H9	119.5	O2—S1—O1	119.85 (10)
C8—C9—H9	119.5	O2—S1—N1	106.49 (10)
C11—C10—C9	120.1 (3)	O1—S1—N1	106.91 (9)
C11—C10—H10	119.9	O2—S1—C1	106.76 (10)
C9—C10—H10	119.9	O1—S1—C1	107.68 (10)
C10—C11—C12	121.6 (3)	N1—S1—C1	108.79 (9)
C10—C11—H11	119.2		
			
C6—C1—C2—C3	0.5 (3)	C10—C11—C12—C7	−2.6 (4)
S1—C1—C2—C3	−177.81 (18)	C10—C11—C12—C15	176.0 (3)
C1—C2—C3—C4	0.3 (4)	C8—C7—C12—C11	3.8 (3)
C2—C3—C4—C5	−1.3 (4)	N1—C7—C12—C11	−179.8 (2)
C2—C3—C4—C13	177.5 (3)	C8—C7—C12—C15	−174.7 (2)
C3—C4—C5—C6	1.5 (4)	N1—C7—C12—C15	1.7 (3)
C13—C4—C5—C6	−177.3 (3)	C8—C7—N1—S1	−103.9 (2)
C2—C1—C6—C5	−0.3 (3)	C12—C7—N1—S1	79.6 (2)
S1—C1—C6—C5	178.06 (18)	C7—N1—S1—O2	−157.30 (17)
C4—C5—C6—C1	−0.8 (4)	C7—N1—S1—O1	−28.06 (19)
C12—C7—C8—C9	−2.0 (3)	C7—N1—S1—C1	87.97 (18)
N1—C7—C8—C9	−178.4 (2)	C6—C1—S1—O2	−26.8 (2)
C12—C7—C8—C14	176.2 (2)	C2—C1—S1—O2	151.58 (18)
N1—C7—C8—C14	−0.2 (3)	C6—C1—S1—O1	−156.66 (17)
C7—C8—C9—C10	−1.1 (4)	C2—C1—S1—O1	21.7 (2)
C14—C8—C9—C10	−179.3 (3)	C6—C1—S1—N1	87.81 (19)
C8—C9—C10—C11	2.3 (5)	C2—C1—S1—N1	−93.86 (19)
C9—C10—C11—C12	−0.4 (5)		

### Hydrogen-bond geometry (Å, °)

**Table:**

*D*—H···*A*	*D*—H	H···*A*	*D*···*A*	*D*—H···*A*
N1—H1N···O1^i^	0.85 (1)	2.20 (1)	3.040 (2)	169 (2)

Symmetry codes: (i) *x*−1, *y*, *z*.

## References

[bb1] Gelbrich, T., Hursthouse, M. B. & Threlfall, T. L. (2007). *Acta Cryst.* B**63**, 621–632. 10.1107/S010876810701395X 17641433

[bb2] Gowda, B. T., Foro, S., Babitha, K. S. & Fuess, H. (2008). *Acta Cryst.* E**64**, o1691. 10.1107/S1600536808024653 PMC2960643 21201680

[bb3] Gowda, B. T., Foro, S., Nirmala, P. G. & Fuess, H. (2010). *Acta Cryst.* E**66**, o144. 10.1107/S1600536809053057 PMC2980205 21580035

[bb4] Gowda, B. T., Foro, S., Nirmala, P. G., Terao, H. & Fuess, H. (2009). *Acta Cryst.* E**65**, o1219. 10.1107/S1600536809016377 PMC2969678 21583088

[bb5] Oxford Diffraction (2009). *CrysAlis CCD* and *CrysAlis RED* Oxford Diffraction Ltd, Yarnton, England.

[bb6] Perlovich, G. L., Tkachev, V. V., Schaper, K.-J. & Raevsky, O. A. (2006). *Acta Cryst.* E**62**, o780–o782.

[bb7] Sheldrick, G. M. (2008). *Acta Cryst.* A**64**, 112–122. 10.1107/S0108767307043930 18156677

[bb8] Shetty, M. & Gowda, B. T. (2005). *Z. Naturforsch. Teil A*, **60**, 113–120.

[bb9] Spek, A. L. (2009). *Acta Cryst.* D**65**, 148–155. 10.1107/S090744490804362X PMC2631630 19171970

